# Resource allocation for emergency teams in Danish Emergency Departments

**DOI:** 10.1186/1757-7241-20-S2-P43

**Published:** 2012-04-16

**Authors:** Thomas Lafrenz, Søren Ø Lindberg, Jeppe Lerche la Cour, Lars Folkestad, Peter Hallas, Mikkel Brabrand

**Affiliations:** 1 Department of Anaesthesiology, Sydvestjysk Sygehus Esbjerg, Denmark; 2 Department of Cardiology, Research Unit 4210, University Hospital Gentofte, Denmark; 3 Department of Clinical Physiology and Nuclear Medicine, University Hospital Glostrup, Denmark; 4 Department of Endocrinology, Sydvestjysk Sygehus Esbjerg, Denmark; 5 Department of Anaesthesiology, Rigshospitalet, Juliane Marie Centeret 4013, Denmark; 6 Medical Admission Unit 272, Sydvestjysk Sygehus Esbjerg, Denmark

## Background

The use of designated emergency teams for cardiac arrest and trauma patients are widely implemented. The use of designated teams in Danish emergency departments has not been investigated.

Our aim was to investigate the use and staffing of designated emergency teams in Danish emergency departments.

## Methods

A cross sectional, questionnaire study was sent to the department chairs of all 20 Danish emergency departments. The four level one trauma centres were excluded.

## Results

Nineteen of twenty (95%) departments responded. Three departments were excluded due to incomplete data.

All departments (n=16) received unselected critically ill patients, patients in cardiac arrest and trauma patients. In 16 (100%) departments there were a designated team that responded to cardiac arrest and trauma patients. Only 5 (31%) departments had access to a designated medical emergency team in the emergency department.

The cardiac arrest teams consisted of median 6,2 (range 5-10) different personnel groups. Of these, 3 (range 1-6) were physicians with only 0,9 (range 0-2) being senior consultants. The other summoned personnel groups included emergency department nurses, nurse anaesthetists, bioanalysts and porters.

The trauma teams consisted of median 9,3 (range 7-11) different personnel groups. Of these, 4,3 (range 2-6) were physicians where 2,6 (range 2-4) were senior consultants. The other summoned personnel groups included emergency department nurses, nurse anaesthetists, radiographers, bioanalysts, and porters.

In 4 (25%) departments there were not access to a medical emergency team, and in 5 (31%) an ad hoc team was created by the emergency department personnel. In 2 (14%) departments a team was created by the attending emergency physician. The staffing of the medical emergency teams relied on patient specific diagnosis, symptoms and triage scores.

## Conclusion

Designated teams for patients in cardiac arrest and trauma patients are widely available in Danish emergency departments. More senior staff is included in trauma teams compared to cardiac arrest teams. In addition few emergency departments in Denmark have designated teams for the reception of unselected critically ill patients. This indicates that emergency care of critically ill patients could be improved.

